# Explanatory Factors for Periprosthetic Infection in Total Knee Arthroplasty

**DOI:** 10.3390/jcm10112315

**Published:** 2021-05-26

**Authors:** Alberto Delgado-González, Juan José Morales-Viaji, Guillermo Criado-Albillos, Adoración del Pilar Martín-Rodríguez, Josefa González-Santos, Remedios López-Liria, Carla Collazo-Riobo, Raúl Soto-Cámara, Jerónimo J. González-Bernal

**Affiliations:** 1 Traumatology and Orthopedic Surgery Department, Burgos University Hospital, 09006 Burgos, Spain; alberto.delgado.med@gmail.com (A.D.-G.); jmoralesvi@saludcastillayleon.es (J.J.M.-V.); gcriadoa@saludcastillayleon.es (G.C.-A.); amartinro@saludcastillayleon.es (A.d.P.M.-R.); 2 Department of Health Sciences, University of Burgos, 09001 Burgos, Spain; ccollazo@ubu.es (C.C.-R.); jejavier@ubu.es (J.J.G.-B.); 3 Department of Nursing Science, Physiotherapy and Medicine, Hum-498 Research Team, Health Research Centre, University of Almería, 04120 Almería, Spain; rll040@ual.es

**Keywords:** patella, total knee arthroplasty, infection

## Abstract

There are many studies whose results reveal possible risk factors for developing an infection after a total knee arthroplasty (TKA). The objective of this study is to analyse the risk factors that depend on the hospital and, especially, if the patellar replacement influences the appearance of periprosthetic infection. A retrospective study was performed, where data from the electronic registry of patients of people over 18 and who had undergone TKA, between the years 2015 and 2018, were reviewed. Dependent variables on the patients and the health care system were collected. The possible associations between the factors and the appearance of infection after TKA were studied using univariate and multivariate regression analyses. A total of 907 primary knee arthroplasties were included in the study. Those patients who had their patella replaced had a significantly higher risk of developing an infection (OR 2.07; 95% confidence interval 1.01–6.31). Likewise, patients who underwent surgery by surgeons with more than 10 years of experience were more than twice as likely to become infected than those operated on by younger surgeons (OR 2.64; 95%CI 1.01–6.97). Male patients were also found to be three times more likely to be infected than women (OR 2.99; 95%CI 1.32–5.74). Those interventions that were longer had a higher risk of infection. The same happened with patients who stayed in the hospital for a longer period of time. The rest of the variables did not show statistically significant results. In this study, it was found that the replacement of the patella may be a factor of infection, but it should be corroborated with randomized clinical trials. Furthermore, patients who underwent longer surgeries or those with prolonged hospital stays should be closely monitored to detect infection as soon as possible and establish the most appropriate treatment.

## 1. Introduction

Osteoarthritis is one of the most prevalent joint diseases, with great impact on the life quality of the patient. In 2010, it was estimated that about 210 million people were affected by this pathology [1]. Osteoarthritis in the hip and knee joints causes greater social expense and greater associated disability than degenerative changes in other joints [2]. Therefore, the treatment of this pathology must be meticulous, starting with conservative treatment, such as weight loss, basic analgesia, or low intensity physical exercise, considering that total knee arthroplasty is the last solution to turn to. Howewer, in cases of very advanced osteoarthritis, TKA can even be considered as the first option [3].

All this makes TKA one of the most common surgeries in developed countries. The number of TKAs performed is expected to rise from now on due to the increasing life expectancy of the population. An increment of up to 3.5 million TKAs is estimated for the year 2030 in the United States—eight times more than those done in 2005 [4]. An increase of around 600% is also foreseen for the year 2030 in prosthetic replacement surgeries [5]. All this means that when we face a patient with significant functional demands and matters of quality of life, suffering a complication during this type of surgery will mean higher healthcare costs. 

Consequently, the increase in prosthetic replacement surgeries has seen a rise in the number of periprosthetic infections. Therefore, it is essential to minimize this type of situation by trying to identify any risk factor (RF) that could lead to an infection.

The infection rate for primary knee arthroplasty is estimated to be between 0.5 and 2.0% [6], being higher in cases of immunosuppression, diabetes mellitus (DM), obesity, intravenous drug use, chronic corticosteroid therapies, and systemic skin disorders, among others [7,8]. Many RF have been identified over the years [9,10], but more research is needed to bring the infection rate as close to zero as possible. Among these RF, we can differentiate between those which depend on the patient, such as DM, obesity, and autoimmune diseases, etc; and those which depend on hospital workers, such as surgical time [4,11], changing gloves [12], or taking antiseptic measures, for example.

Depending on the type of prosthesis, there has been a certain tendency to have a higher infection rate for total arthroplasties compared to unicondylar ones. So we would have to say that, as postulated in the second international consensus on musculoskeletal infections [13], a smaller prosthesis may represent also a smaller substrate for microbial colonization. Although no conclusive studies have been conducted linking this in theory, replacing the patella implies an increase in the prosthetic surface that can end up with an infection, but more detailed studies need to be performed. Therefore, it could be thought that patellar replacement is another RF in terms of developing a periprosthetic infection. This has been a controversial question over the years. Those who defend the systematic replacement of the patella do so based on certain studies that report a greater presence of anterior knee pain after arthroplasty in those who did not have the patella replaced [14,15,16]. While those who advocate not replacing it do so based on higher rates of patellar fracture in the cases when this bone was replaced, in addition to possible changes in patellar height [17,18]. A meta-analysis carried out in 2018 that included six articles referring to the infection concluded that there were no differences between the replacement of the patella or not [19]. This study aims to identify risk factors associated with infection after a TKA.

## 2. Materials and Methods

### 2.1. Design–Study Population

Descriptive retrospective study, in which data from the electronic registry of the Burgos University Hospital (Burgos, Spain) were reviewed. This is a third-level health centre, with Traumatology and Orthopaedic Surgery Service, which is a regional reference for revision arthroplasty.

The inclusion criteria of the study were: patients over 18, admitted to the Orthopaedic Service of the Burgos University Hospital, between 1 January 2015 to 31 December 2018; who, on a scheduled basis, underwent a TKA. No exclusion criteria were considered. 

The study’s research protocol was approved by the Ethical Committee for Clinical Research with Medicines of the Burgos and Soria Health Area (CEIm-2230), meeting at all times the requirements of the 1975 Helsinki Declaration. Informed consent signatures were not required.

### 2.2. Data Collection–Outcomes Measures

Data were collected after analysis of electronic consultations and followed by manual reviews of electronic medical records. The mean follow-up period after surgery was 2.3 years (SD 0.6) and the minimum and maximum were 1.5 and 3 years, respectively.

The main outcome variable of this study was the development of infection in the prosthetic knee. The diagnosis was made by germ isolation in a synovial fluid sample [20]. Electronic medical records were reviewed by members of the clinical research team for clinician diagnoses of post-operative infections.

The possible predictive variables were classified as dependent on the patient (sex, age, personal history of high blood pressure or DM, anaesthetic risk, and laterality) and dependent on the healthcare system (start time and duration of surgery, trademark of the prosthesis, replacement of the patella by prosthetic implant, previous experience of the surgeon, presence of medical personnel in specialized training, placement of drainage or bladder catheterization, and days of hospital stay) [21,22]. Patients were considered to be hypertensive if they had systolic (SBP) or diastolic blood pressure (DBP) of more than 140 mmHg or 90 mmHg, respectively, and/or were taking antihypertensive drugs. DM was defined as a fasting plasma glucose level higher than 7.00 mmol/L, glycosylated haemoglobin higher than 6.5%, and/or taking oral antidiabetic drugs and/or insulin. Medical personnel in specialized training were doctors who have obtained a degree in medicine and surgery, but have still specialized in orthopaedics and traumatology, which consists of a training period of five years.

Anaesthetic risk was assessed using the scale proposed by the American Society of Anesthesiologists (ASA), which classifies patients into five levels, based on their health status [23]. The start time of the surgery was classified into two groups: before and after 11:00 am, in such a way that the procedures performed after the chosen cut-off point were always preceded by a previous surgical intervention [24]. To evaluate the duration of the surgery, the time elapsed from its onset was defined as the moment the patient entered the operating room until the moment they left. The cut-off point for dichotomizing the surgeon’s previous experience was 10 years [25,26,27]. The infection time was defined as the days elapsed from the implantation of the prosthesis to the establishment of the infection diagnosis as previously indicated in this article. The assignment of the patient to a specific surgeon was done randomly without regard to the characteristics of the patient or the surgeon.

### 2.3. Statistical Analysis

For the characterization of the sample, absolute frequencies and percentages were used if the variables were categorical or the mean and standard deviation (SD), or the median and interquartile range (IQR) in the case of quantitative variables, depending on the type of distribution they followed. To determine the association between infection of the total knee prosthesis with the categorical independent variables, the chi square test or Fisher’s exact test was used; while for comparison with the independent quantitative variables, the Student-*t* test was performed for independent samples and one-way ANOVA if they followed a normal distribution or the Mann-Whitney U test or the Kruskal-Wallis test if they didn’t follow it. To quantify the magnitude of the association between variables, the odds ratio (OR) was calculated using a forward selection multivariate regression analysis, adjusted for sex and age, which included all those variables that obtained statistical significance in the previously univariate analysis. The existence of statistical significance was considered if *p* < 0.05. Statistical analysis was performed with SPSS version 25 software (IBM SPSS Inc, Chicago, IL, USA).

## 3. Results

A total of 907 patients were included in the study, with a homogeneous distribution over gender and age. 43.00% (*n* = 400) of the operated patients were males. The mean age was 71.40 years (SD ± 8.19), being significantly higher in women (72.63 ± 7.98 versus 69.86 ± 8.21; *p* < 0.001). Three out of five and one in five patients had a personal history of high blood pressure and DM, respectively, both pathologies being more prevalent in older patients. The anaesthetic risk assessment, performed using the scale proposed by the ASA, revealed that it was low (I–II) in 80.2% of the patients. 71.44% (*n* = 648) of the interventions started before 11:00 am, with an average duration of 130.10 minutes (SD ± 23.47). Two thirds of surgeons had previous experience of more than 10 years, being supported at all times by medical personnel in training with different degrees of specialization. The patella was replaced by a prosthetic implant in 278 patients related to the female sex (*p* < 0.001), with a higher mean age of the patient (*p* < 0.001), with the longest mean duration of surgery (*p* < 0.001), with not performing ischemia during the intervention (*p* < 0.001), with a previous experience of the surgeon of more than 10 years (*p* = 0.003) or with the placement of drainage (*p* < 0.001). The patients were discharged after a mean admission period of 6.48 days (SD ± 2.57). Infection of the total knee prosthesis occurred in 33 out of 907 cases (3.6%): the mean time of onset of this situation was four weeks, with an IQR that ranged from 0.68 to 14.25 weeks.

Male sex was the only patient-dependent factor that was significantly related to infection of the total knee prosthesis (*p* = 0.005). Among the factors most directly linked to professionals or the health system were the experience of the surgeon over 10 years (*p* = 0.023), the replacement of the patella by a prosthetic implant (*p* = 0.049), a longer duration of surgery (*p* = 0.032), or a longer post-surgical hospital stay of the patient (*p* = 0.002) (Table 1). The median time of onset of infection in weeks was higher in women (11.00, IQR 3.00–24.00 versus 1.00, IQR 0.58–6.50; *p* = 0.050) and in patients who were carriers of a drainage after surgery (11.50, IQR 3.75–21.75 versus 0.87, IQR 0.50–4.00; *p* = 0.002), not related to the rest of the analysed factors.

In the multivariate analysis performed (forward selection), with the significant factors from Table 1, the factors that were related to a greater probability of developing a post-surgical infection after TKA were: male sex (OR 2.99; 95% confidence interval (CI) 1.32–5.74), previous experience of the surgeon over 10 years (OR 2.64; 95% CI 1.01–6.97), or replacement of the patella by a prosthetic implant (OR 2.07; 95% CI 1.01–6.31) (Table 2).

## 4. Discussion

The most important finding of this study could be the association between patellar replacement and the possibility of developing an infection. This study analyses the RFs associated with the development of a total knee prosthesis infection in patients undergoing a scheduled replacement of the knee joint, some of which have not been previously individually assessed. The results obtained reveal that the infection rate of the total knee prosthesis is slightly higher than accepted, affecting four out of 100 patients. Some of the influencing factors in our study depend on the healthcare system, while only one depends on the patient.

In our study, male sex is the only patient-dependent factor that has been associated with an increased risk of developing an infection in the TKAs, becoming up to three times higher. It has been theorized that this may be due to increased tissue hypoxia, skin thickness, or different bacterial colonization, and there is no clear conclusion in this regard [22]. On the other hand, in this study, the age of the patient has not been a predisposing factor for the appearance of infection, a result which is contrary to that observed by other authors in other studies, where the older the patient was, the higher the infection rate was, too. The same happens with other factors dependent on the patient, such as the presence of DM or high blood pressure [22].

Regarding the factors dependent on the health system, the experience of the surgeon and the replacement of the patella were the ones which significantly increased the risk of developing an infectious process. One of the main findings of the present study is that patients who undergo prosthetic replacement of the patella have twice the risk of developing infection in relation to those other cases in which it was not replaced (*p* = 0.046). Although the significance was small, that information was not observed in any previously conducted study. Those studies that reported the infection rate according to whether the patella was replaced or not, obtained the majority of infections in those patients who had the patella replaced, but without being statistically significant. Although it is important to note that these studies reported infection as a complication of surgery, they did not conclude if it was a consequence of patellar replacement [28,29,30,31,32] or not. These randomized studies indicate that no general recommendations can be made in this regard. It is necessary for more randomized clinical trials to establish if the causal relationship between patella replacement and the appearance of infection in knee arthroplasties exists. Considering our results, the patella should not be replaced systematically, but rather in selected patients appropriately, as doing so may increase the risk of infection and not all patients may need a prosthetic patella. When it comes to the experience of the surgeon, some literature describes that although greater experience might be considered a benefit, there is an inverse and paradoxical relationship between it and the quality of patient outcomes [33]. Substantial numbers of aging surgeons will bear greater workloads and their level of stress would increase [34]. Other factors that may be involved in surgeon experience relate to the operating room environment (teaching hospital or hospital volume) [35]. In our opinion, more aged surgeons used to perform more complex cases than younger surgeons, and this could also explain a higher prevalence of infection; while at teaching hospitals more personnel are involved (anesthesia and orthopedic residents, medical students, educational visitors, young nurses, and specialists to manage instruments and modular implants).

The duration of the surgery or the days of hospital stay have been related to an increase in the infection rate, which may be due to the patient being in an aggressive and potentially dangerous environment for him or her [22]. However, in our study, this influence disappears when analysed together with other factors. Contrary to what was considered in other studies, in which the duration of the surgery is defined as the time interval that extends from the first incision in the skin to the complete closure of the wound [4,14], in this study it has been considered as the interval that elapses from when the patient enters the operating room until leaving it, due to possible iatrogenesis of the environment. An important point to keep in mind is to try to minimize surgical time and average hospital stay, closely monitoring those patients for whom this situation has not been possible. So those patients with very long interventions or long stays should be followed up more exhaustively in order to detect possible infection as soon as possible and to establish the best existing treatment.

Despite the fact that in the literature an increase in the infection rate has been described over the years in those patients who carried a drainage, especially if it was for a long time, possibly related to a greater need for blood transfusion, in our study we did not observe differences between these groups of patients, although we could conclude that over the years they were used less and less frequently [36,37]. Something similar happened in the case of bladder catheterization, a practice that has also been related to a possible increase in the infection rate but in our study no significant differences were observed, although, again, there was a tendency to use them less and less [11].

As limitations in the study, we mainly consider its retrospective nature and that it was only performed in one hospital centre. The size of the sample could be considered small (it was confined to a Spanish population) and that could be significant. Furthermore, the variables were those already existing in the database, without the ability to add or modify any. For example, the surgery duration was defined as the elapsed time from the moment the patient entered the operating room until the moment they left, instead the elapsed time between the incision and skin closure. In future studies it would also necessary to consider the relationship between surgical experience and patellar replacement rate, and the length of hospitalization (a longer hospitalization should be evaluated for more factors).

On the other hand, we consider the strengths to be that the population was representative of the current clinical practice, and without losses in the study for four consecutive years. There was no manipulation of the patient’s assignment or the techniques used and we considered that the procedure of the study did not significantly affect the results.

The identification of the most important RFs is a key element to implement measures that could reduce the rate of infection after TKA. Effective strategies to minimize RF identified in this paper and previous studies must be stringently instituted following the perioperative protocols. In our study, it was found that for male patients having replacement of the patella, longer intervention and hospital stay may be RFs of infection, but these factors should be corroborated with randomized clinical trials. Although it is not possible to draw absolute conclusions from this study, it must be valid to establish the bases for future studies on the subject. These results allow us to establish some preventive measures of TKA, such as surgery performed or operative time reduction, in addition to those already established in general terms, such as antibiotic prophylaxis and skin preparation.

## 5. Conclusions

Determining the RFs for the development of an infection in TKA is crucial to try to reduce this complication as much as possible. 

In our study, we observed that certain risk factors could predispose to developing a possible prosthetic infection; such as male sex, increased surgical time, or a prolonged hospital stay. All of these factors are widely described in the literature. Perhaps the most important fact that emerges from our study is the possible association of the infection with the patella resurfacing, which should be individually assessed. This should be corroborated in future randomized clinical trials.

## Figures and Tables

**Table 1:** Comparison of the characteristics of TKA depending on the development or not of post-surgical infection.

	Infection		*p*-Value
	Yes	No	
Patient-dependent factors			
Age-*X (DS)*	70.33 (8.17)	71.49 (8.19)	0.432
Gender-*n* (%)			
Male	22 (2.43)	368 (40.57)	0.005
Female	11 (1.21)	506 (55.79)	
High blood pressure-*n* (%)			
Yes	21 (2.32)	517 (57.00)	0.607
No	12 (1.32)	357 (39.36)	
Diabetes mellitus-*n* (%)			
Yes	9 (0.99)	169 (18.63)	0.26
No	24 (2.65)	705 (77.73)	
ASA classification-*n* (%)			
I–II	23 (2.54)	704 (77.62)	0.125
III–IV–V	10 (1.10)	170 (18.74)	
Side-*n* (%)			
Right	18 (1.99)	452 (49.83)	0.75
Left	15 (1.65)	422 (46.53)	
Dependent factors of the health system			
Time of the surgery-*n* (%)			
Before 11:00 a.m.	27 (2.98)	621 (68.47)	0.179
After 11:00 a.m.	6 (0.66)	253 (27.89)	
Brand of the prosthesis-*n* (%)			
Hifit^®^	15 (1.65)	343 (37.82)	0.238
Nexgen^®^	2 (0.22)	152 (16.76)	
Others	16 (1.76)	379 (41.79)	
Patella substitution-*n* (%)			
Yes	15 (1.65)	263 (29.00)	0.049
No	18 (1.99)	611 (67.36)	
Surgeon experience-*n* (%)			
≤10 years	5 (0.55)	299 (32.96)	0.023
>10 years	28 (3.09)	575 (63.40)	
Resident experience-*n* (%)			
1st year	9 (0.99)	164 (18.08)	0.174
2nd year	12 (1.32)	230 (25.36)	
3rd year	2 (0.22)	141 (15.55)	
4th year	2 (0.22)	136 (15.00)	
5th year	8 (0.88)	203 (22.38)	
Use of drainage-*n* (%)			
Yes	21 (2.32)	517 (57.00)	0.607
No	12 (1.32)	357 (39.36)	
Use of urinary catheter-*n* (%)			
Yes	9 (0.99)	159 (17.53)	0.26
No	24 (2.65)	705 (77.73)	
Surgery duration-*X (DS)*	136.67 (16.99)	129.85 (23.65)	0.032
Days of hospital stay-*X (DS)*	8.18 (7.10)	6.79 (2.22)	0.002
Year-*n* (%)			
2015	6 (0.66)	247 (27.23)	0.277
2016	7 (0.77)	206 (22.72)	
2017	11 (1.21)	177 (19.52)	
2018	9 (0.99)	244 (26.90)	

ASA: American Society of Anesthesiologists; X: Mean; DS: Standard deviation.

**Table 2:** Multivariate regression analysis of predictive factors for post-surgical infection after TKA.

	Odds Ratio (OR)	Confidence Interval 95%	*p*-Value
Gender: Male	2.99	1.32–5.74	0.004
Patella substitution: Yes	2.07	1.01–6.31	0.046
Surgeon experience: >10 years	2.64	1.01–6.97	0.050
